# Integrative visualization of the molecular structure of a cellular microdomain

**DOI:** 10.1002/pro.4577

**Published:** 2023-02-14

**Authors:** David S. Goodsell, Keren Lasker

**Affiliations:** ^1^ Department of Integrative Structural and Computational Biology The Scripps Research Institute La Jolla California USA; ^2^ Research Collaboratory for Structural Bioinformatics Protein Data Bank, Rutgers, The State University of New Jersey Piscataway New Jersey USA; ^3^ Institute for Quantitative Biomedicine, Rutgers, The State University of New Jersey Piscataway New Jersey USA; ^4^ Rutgers Cancer Institute of New Jersey, Rutgers, The State University of New Jersey New Brunswick New Jersey USA

**Keywords:** *Caulobacter crescentus*, cellular condensate, integrative structural biology, intrinsically disordered protein, molecular visualization, PopZ

## Abstract

An integrative approach to visualization is used to create a visual snapshot of the structural biology of the polar microdomain of *Caulobacter crescentus*. The visualization is based on the current state of molecular and cellular knowledge of the microdomain and its cellular context. The collaborative process of researching and executing the visualization has identified aspects that are well determined and areas that require further study. The visualization is useful for dissemination, education, and outreach, and the study lays the groundwork for future 3D modeling and simulation of this well‐studied example of a cellular condensate.

## 1 INTRODUCTION

**FIGURE 1:**
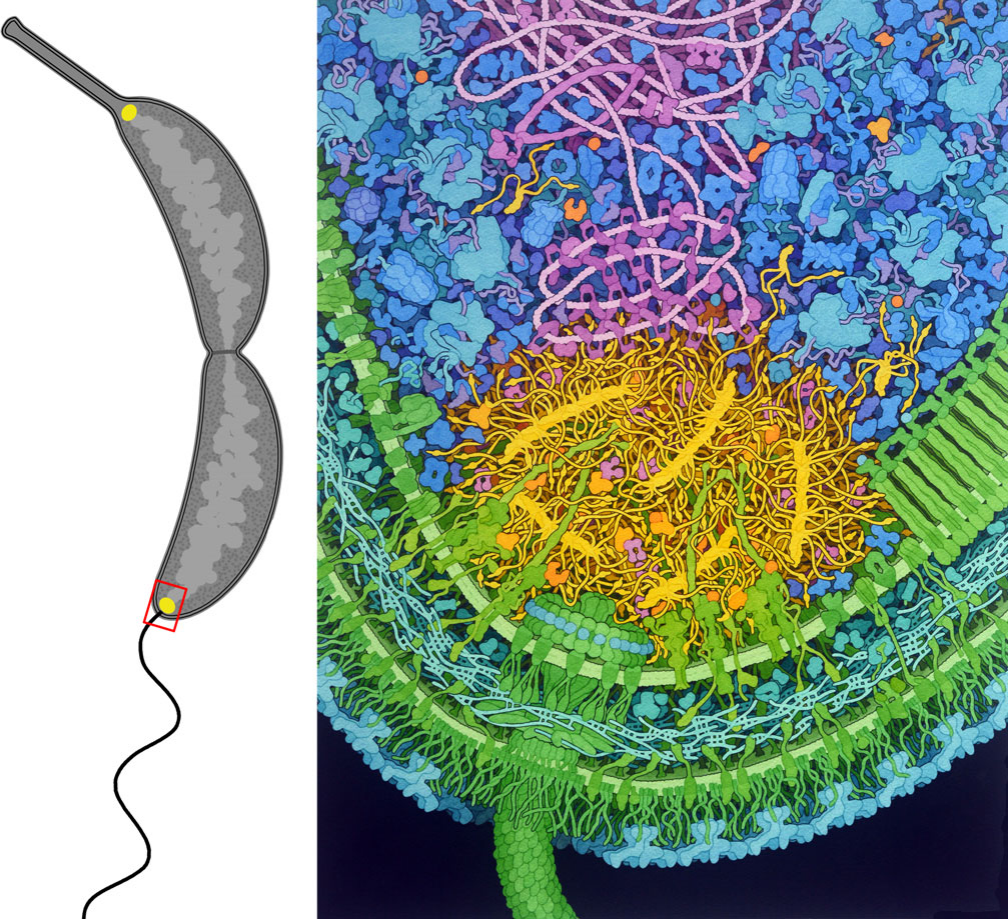
Illustration of a predivision *Caulobacter* cell (left) with the sessile daughter cell at the top and the swarmer daughter cell at the bottom. Close‐up of the flagellated pole (right), with the PopZ in yellow, soluble clients in orange, cytoplasm in blue and purple, nucleoid in magenta, and cell wall in green and turquoise.

The aquatic bacterium *Caulobacter crescentus* has emerged as a simple model organism for study of cellular development and differentiation (Curtis & Brun, 2010; Govers & Jacobs‐Wagner, 2020; Lasker et al., 2016; Tsokos & Laub, 2012). It has a two‐step life cycle, born as a DNA replication‐incompetent free‐swimming “swarmer” and differentiating into a DNA replication‐competent immobile “stalked” cell. As part of this process, the cell populates the two ends of the cell (poles) with scaffolding and signaling proteins that regulate these two distinct life forms (Figure 1, left). These specialized polar complexes guide construction of flagellar motors and pili in the swarmer form and the shedding of the flagellum, retraction of the pili, and construction of a stalk with an adhesive tip (holdfast) in the stalked form (Ardissone & Viollier, 2015; Mignolet et al., 2018). Critically, these complexes regulate signaling pathways that activate replication and segregation of the DNA chromosome exclusively in stalked cells (Frandi & Collier, 2019).

How are these signaling proteins localized to the pole and how is the composition of the polar region maintained? These polar regions are defined by self‐assembly of the polar organizing protein Z (PopZ) that marks a specific microdomain at the poles, selectively gathers scaffolding and signaling macromolecular complexes (“client” proteins), and excludes large cytoplasmic molecules like ribosomes as well as smaller proteins that do not bind to members of the microdomain (Bowman et al., 2008, 2010; Ebersbach et al., 2008; Gahlmann et al., 2013; Holmes et al., 2016; Lasker et al., 2020). In this illustration (Figure 1, right), we capture the current knowledge about the composition and structure of the PopZ microdomain at the flagellated pole. In particular, we highlight the role PopZ plays in bringing these signaling proteins together. Below, we provide a brief overview of the main components of this illustration, namely PopZ and the clients that reside within this microdomain.

### 1.1 PopZ, clients, and the polar microdomain

**FIGURE 2:**
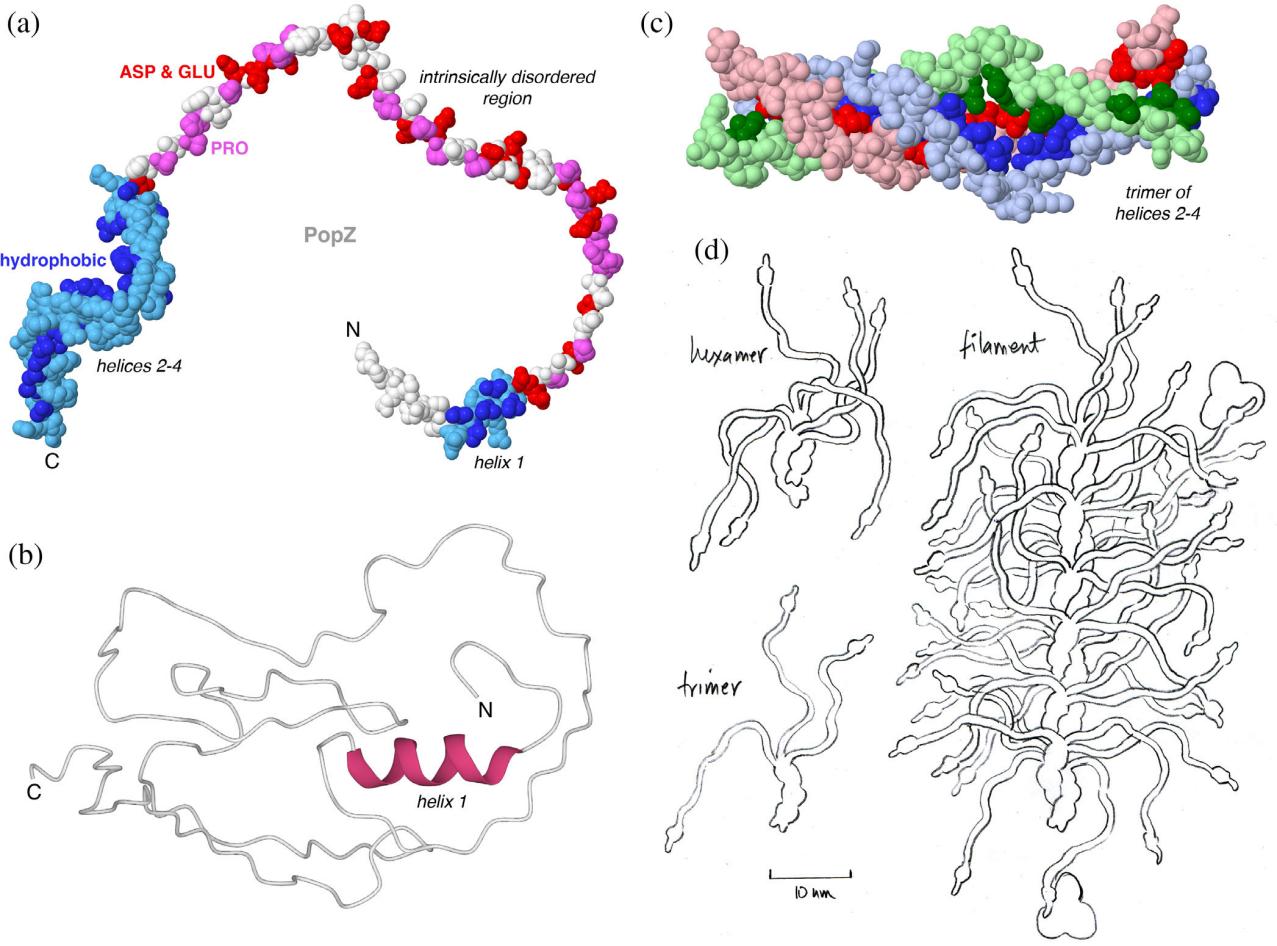
PopZ structures and models. (a) Model of the PopZ monomer as predicted by AlphaFold2 (top) contains a short N‐terminal helix (blue), an IDR rich in proline (magenta) and negatively charged amino acids (red), and three closely spaced helices at the C‐terminus (blue). The helices are amphipathic, as illustrated by coloring hydrophobic amino acids a darker shade of blue. The N‐terminal helix interacts with client proteins and the C‐terminal helices mediate oligomerization. Coordinates for PopZ from AF‐Q9A8N4‐F1‐model_v3, with modified torsion angles at residue 73 to give a more extended structure for this figure. (b) Structure of PopZ 1–133 determined by NMR, from PDB ID 6xry, colored by secondary structure with alpha helices in magenta and disordered regions in white. (c) Trimer model of C‐terminal region predicted using AlphaFold2 (manuscript in preparation). (d) Sketch of PopZ trimer, hexamer, and filament based on these structures, created during the collaborative dialog between the authors.

PopZ shows many of the characteristic features shared by molecules that form cellular condensates (Banani et al., 2017; Nordyke et al., 2020; Posey et al., 2018; Ptacin et al., 2014; Shin & Brangwynne, 2017). PopZ is largely disordered. Its N‐terminal region binds clients through a short conserved helix (Holmes et al., 2016; Nordyke et al., 2020). The central region of PopZ is disordered with many prolines and negatively charged amino acids and likely acts as a tuner of PopZ material properties (Lasker et al., 2022). Self‐assembly is mediated by interactions between three helices at the C‐terminal helical region (Figure 2a,b; Bowman et al., 2010; Bowman et al., 2013; Laloux & Jacobs‐Wagner, 2013; Lasker et al., 2022). In our current model, based on biochemical and structural data, PopZ associates into trimers and hexamers decorated with long intrinsically disordered regions (IDRs), with client‐interacting helices at their tips (manuscript in preparation). The hexamers are likely to further associate into filaments, as observed in cryoelectron micrographs (Toro‐Nahuelpan et al., 2022; Figure 2c,d).

The PopZ condensate recruits multiple client proteins that mediate pole‐specific functions in the cell. Currently known clients are enumerated in Table 1, and include cytosolic regulatory proteins and a diverse collection of membrane‐bound histidine kinases, scaffolding proteins, and proteins that mediate formation of the flagellar motor and pili. Clients that are specific to the stalk pole are not included in this list.

**TABLE 1:** Molecular oligomerization and abundances.

Protein	Assembly	Number/cell^a^	Fraction^b^	Number/foreground^c^	Reference
PopZ	6	8200	0.5	28.5	(Bowman et al., 2008; Ebersbach et al., 2008)
Membrane clients					
Podj	2	700	0.9	8.8	(Hinz et al., 2003; Viollier et al., 2002)
PleC	2	688	0.8	7.6	(Jacobs et al., 2001)
DivL	2	589	0.6	4.9	(Tsokos et al., 2011)
CckA	2, 4^d^	734	0.8	8.2	(Angelastro et al., 2010)
ZitP	1	617	0.9	15.4	(Bergé et al., 2016)
Soluble clients					
ChpT	2	1493	0.2	6.2	(Guzzo et al., 2021; Lasker et al., 2020)
CtrA	2	25,402	0.03	15.9	(Lasker et al., 2020; Ryan et al., 2004)
CpdR	1	6044	0.01	2.5	(Iniesta et al., 2006)
RcdA	2	2583	0.03	1.6	(McGrath et al., 2006)
ParB	2	2072	0.45	19.4	(Jalal & Le, 2020; Ptacin et al., 2014)
DivK	1	2671	0.03	3.3	(Matroule et al., 2004)
PleD	2	1359	0.03	0.8	(Paul et al., 2004)
PopA	1	930	0.3	11.6	(Duerig et al., 2009; Wang et al., 2021)

^a^ Number of protein chains per cell.

^b^ Fraction of molecules located at the pole.

^c^ Number of assemblies to be included in the foreground layer of the painting. See text for calculation.

^d^ May assemble into tetramers, as shown in the illustration.

### 1.2 Regulatory cascades

The two‐component signaling protein CtrA is the main regulator of *Caulobacter* asymmetry through its dual roles as an inhibitor of DNA replication initiation and as a transcription factor that controls expression of many genes involved in swarmer cell fate (Laub et al., 2002; Quon et al., 1998). Unlike other transcriptional regulators in *Caulobacter*, CtrA must be phosphorylated (CtrA~P) to be active as a transcription factor (Domian et al., 1997).

CtrA levels and its phosphorylation state are regulated by signaling cascades that happen primarily at the poles, which implement either a CtrA activation or CtrA degradation cascade depending on cell‐cycle state (Zik & Ryan, 2022). Upon compartmentalization of the predivisional cell, CtrA drives asymmetry by remaining phosphorylated and active in the swarmer compartment while being dephosphorylated and degraded in the stalked compartment (Tsokos & Laub, 2012).

The phosphorylation state and levels of CtrA are determined by the bifunctional hybrid histidine kinase CckA (Jacobs et al., 1999). When stimulated as a kinase, CckA autophosphorylates and transfers its phosphate to the phosphotransfer protein ChpT (Biondi et al., 2006; Blair et al., 2013; Chen et al., 2009), which in turn passes the phosphate to either CtrA, resulting in its activation, or to CpdR, resulting in inhibition of CtrA degradation (Iniesta & Shapiro, 2008). When CckA is stimulated as a phosphatase, phosphoryl groups are transferred back from CtrA through ChpT to CckA, where they are hydrolyzed (Biondi et al., 2006). This reverse process inhibits the activation of CtrA and promotes CtrA degradation by shutting off the flow of phosphate to CpdR. The dual functions of CckA act as a switch that leads to either CtrA activation, to promote swarmer fate, or CtrA degradation, to promote stalk fate.

CckA state is in turn regulated by the composition of the pole it resides in. Accumulation of CckA at the flagellated cell pole is mediated by the pseudokinase DivL and promotes kinase activity in the swarmer compartment (Iniesta & Shapiro, 2008; Mann & Shapiro, 2018; Mann et al., 2016). In addition, the phosphorylation state of two response regulators, DivK and PleD (Hecht et al., 1995; Hecht & Newton, 1995) modulate CckA state. DivK, when phosphorylated, binds to DivL, and this DivL‐DivK~P complex can inhibit CckA kinase activity (Childers et al., 2014; Tsokos et al., 2011). PleD, when phosphorylated, synthesizes cyclic‐di‐GMP (cdG), which can bind CckA and inhibit its kinase activity (Lori et al., 2015; Mann et al., 2016). The levels of DivK~P and PleD~P are modulated by the swarmer fate determinant PleC, which is localized to the flagellated pole and the stalked fate determinant DivJ, which is localized to the stalk cell pole. PleC dephosphorylates DivK‐P and PleD~P, permitting the activity of CckA in the swarmer compartment (Abel et al., 2013; Christen et al., 2010; Lam et al., 2003; Wheeler & Shapiro, 1999). Thus, CckA integrates information about the progression of assembly of the CtrA activation pathway and of cellular compartmentalization to regulate the phosphorylation state and stability of CtrA.

The AAA+ protease ClpXP degrades CtrA in a cell cycle‐dependent manner (Fatima et al., 2022; Joshi & Chien, 2016; Schroeder & Jonas, 2021). CpdR, RcdA, and the cdG receptor PopA are required for degradation of CtrA by ClpXP when bound to the chromosome. The signaling proteins inside the polar region coordinate the availability of these three adaptors, in a hierarchical manner, to bind ClpXP at the right time and place (Gora et al., 2013; Joshi et al., 2015; Smith et al., 2014).

### 1.3 ParB and DNA segregation

The ParABS system is an active DNA segregation system that was first identified to play a role in the stable inheritance of low‐copy‐number plasmids (Abeles et al., 1985; Austin & Abeles, 1983; Mori et al., 1986). In addition to plasmids, numerous bacterial species use ParABS to segregate their chromosomes (Livny et al., 2007), including *C. crescentus* (Mohl et al., 2008; Ptacin et al., 2010). The ParABS system consists of three conserved components: the origin‐proximal centromeric DNA sequence, *parS*; the CTPase ParB, which binds *parS*, spreads, and nucleates a nucleoprotein complex on a *parS* sequence (Antar et al., 2021; Böhm et al., 2020; Breier & Grossman, 2007; Lagage et al., 2016), and the Walker‐box ATPase ParA, which interacts with ParB and drives the segregation of the ParB‐DNA nucleoprotein complex to partition replicated chromosomes to each daughter cell (Easter & Gober, 2002; Leonard et al., 2005). In *Caulobacter*, PopZ anchors the ParB‐*parS* complex to the cell poles through direct interaction with ParB (Bowman et al., 2008). PopZ also binds ParA, and sequestration of ParA into PopZ stimulates recycling of ParA into a nucleoid‐bound complex to ensure pole‐specific centromere transfer (Bowman et al., 2008; Laloux & Jacobs‐Wagner, 2013; Ptacin et al., 2014).

### 1.4 Integrative visualization

This work is based on the hypothesis: “Can we create a visualization that shows a portion of this cell, including all macromolecules and based on the current state of structural, bioinformatic, biochemical, and micrographic knowledge?” We have taken a two‐pronged approach to address this hypothesis in other systems. First, we have used traditional scientific illustration methods to create depictions of cross‐sections through many types of cells, primarily for use in scientific dissemination, education, and outreach (Goodsell, 1991, 2021). Second, when technically possible, we have created 3D computational models of these cells, for example, in recent models of whole mycoplasma and minimal genome cells (Goodsell, 2022; Maritan et al., 2022). Increasingly, we are using the illustration effort as a prelude to full 3D modeling, as a tool to enumerate the many types of structures and interactions that need to be modeled. This report presents the first step in this process for the *Caulobacter* flagellar polar region, gathering and curating the necessary information and integrating it to create an illustrative snapshot of the cell that captures the current state of knowledge (Figure 1).

## 2 RESULTS

### 2.1 Overview of illustration

The illustration depicts the flagellar pole of a *Caulobacter crescentus* predivisional cell, centered on the PopZ microdomain. Each PopZ filament comprised of 5–7 hexamers, with many IDR extending from a central helical core. Helix 1 is shown as a small globule near the end of each IDR. Membrane‐bound and soluble clients are embedded in the PopZ matrix, primarily interacting with Helix 1, and several free soluble clients are shown in the adjacent cytoplasm, along with free PopZ trimers and hexamers. The nucleoid interacts with PopZ through the client protein ParB, which is shown condensing on a short region of the DNA through spreading of closed forms and bridging of open forms (Jalal & Le, 2020). Also shown on the chromosome are the DNA‐binding proteins SMC (Jensen & Shapiro, 1999; Tran et al., 2017) and GapR (Guo et al., 2018) as well as the master transcription factor CtrA bound to five sites near the chromosomal origin of replication. Other features specific to the flagellated pole include a flagellar motor and chemosensory array.

### 2.2 Sources and confidence

**FIGURE 3:**
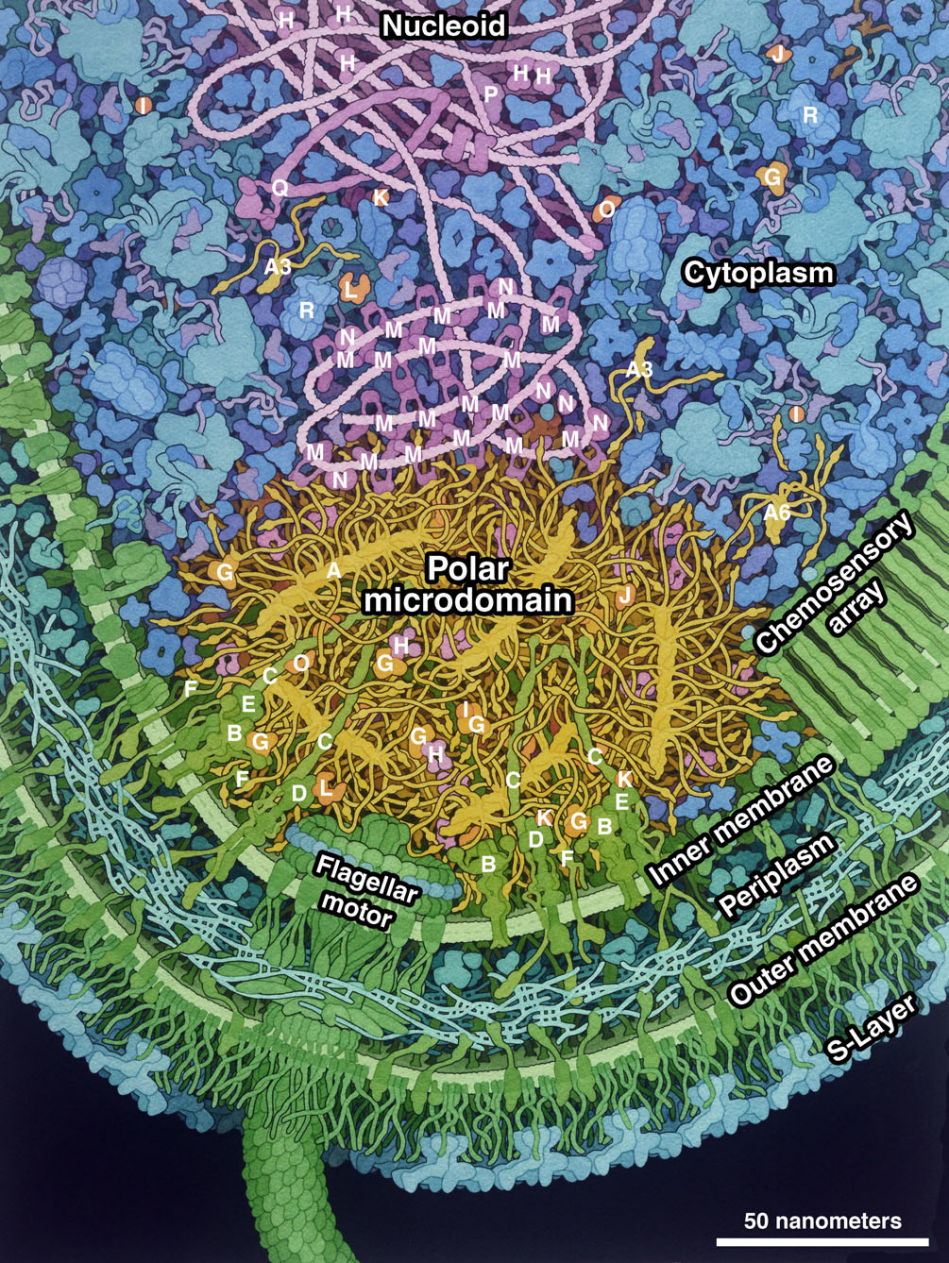
Key to the illustration. PopZ: A, PopZ microfilament; A6, PopZ hexamer; A3, PopZ trimer. Clients: B, CckA; C, PodJ; D, PleC; E, DivL; F, ZitP; G, ChpT; H, CtrA; I, CpdR; J, RdcA; K, DivK; L, PleD; M, ParB; N, MipZ; O, PopA; P, ParA. Proteins related to the microdomain: Q, SMC (structural maintenance of chromosomes); R, ClpXP.

Diverse sources of data were gathered, curated, and integrated into the illustration, as described in detail in the Methods. The ultrastructure is based on cryoelectron tomography and fluorescence microscopy, which is sufficient to define the geometry of the cell wall and chemosensory array, positions of individual ribosomes, and volume of the microdomain (Dahlberg et al., 2020; Gahlmann et al., 2013; Lasker et al., 2020). The flagellar motor is not present in the source micrographs, and was added based on separate single‐particle cryoEM studies (Rossmann et al., 2020). Structures for individual molecules were gathered from multiple sources, leveraging the effective search methods available at UniProt (uniprot.org) and RCSB PDB (rcsb.org). These include experimental structures of *Caulobacter* proteins and proteins from related organisms, homology models, and predicted structures from AlphaFold2 (Jumper et al., 2021). Individual proteins related to the microdomain are identified in Figure 3.

Abundances are based on data from ribosome profiling (Schrader et al., 2016). The fraction of each protein found at the pole is determined based on relative fluorescent levels of the protein at the pole versus the rest of the cell (Table 1). The absolute number of molecules depicted in the painting is estimated using these abundances and fractions, scaled to give an overall concentration similar to the cytoplasm. Interactions were among the most difficult types of information to find: they were determined one‐by‐one by searching the primary research literature and by personal communication with the community of *Caulobacter* experts (Table 2).

**TABLE 2:** Microdomain interactions.

Interacting	Proteins	Reference
PopZ	PodJ	(Zhao et al., 2018)
PopZ	CckA	(Holmes et al., 2016)
PopZ	ZitP	(Bergé et al., 2016)
PopZ	ChpT	(Holmes et al., 2016; Lasker et al., 2020)
PopZ	CpdR	(Holmes et al., 2016)
PopZ	RcdA	(Nordyke et al., 2020)
PopZ	ParB	(Bowman et al., 2008; Ptacin et al., 2014)
PodJ	PleC	(Zhang et al., 2022)
PodJ	DivL	(Curtis et al., 2012)
PodJ	PopA	(Curtis et al., 2012)
PleC	DivK	(Childers et al., 2014; Matroule et al., 2004)
PleC	PleD	(Aldridge et al., 2003)
DivL	CckA	(Childers et al., 2014; Tsokos et al., 2011)
DivL	DivK	(Reisinger et al., 2007; Tsokos et al., 2011)
ZitP	CpaE	(Bergé et al., 2016)
ChpT	CtrA	(Blair et al., 2013)
ChpT	CpdR	(Blair et al., 2013)
ParB	ParA	(Figge et al., 2003)
ParB	MipZ	(Figge et al., 2003)

In these types of integrative efforts, which seek to include all macromolecules in the proper place and proper abundance, it is necessary to work with the current state of knowledge, which may be fragmentary for some aspects of the scene. The *Caulobacter* illustration heavily leverages previous work on *E. coli* for overall features of the cytoplasm and cell wall, while confirming the presence of each molecule in the *Caulobacter* genome. Strong structural data are available for the soluble clients; however, the membrane‐bound clients and PopZ are all subjects of this study, so these molecules are depicted based on speculative models informed largely by predicted models from AlphaFold2 and relationships to similar well‐characterized systems. Interactions between PopZ and clients are also a topic that is currently under investigation—generally, the interaction sites in PopZ are well defined, but the sites on clients less so.

### 2.3 Design and rendering choices

As in previous illustrative work, the *Caulobacter* illustration employs intentional artistic license to improve the readability of the image (Goodsell, 2009, 2022). The image depicts all macromolecules using simplified shapes, all drawn in canonical views for easy recognition. Disordered molecules (PopZ IDR, mRNA, lipopolysaccharide, etc.) are rendered with slightly exaggerating chain width to improve visibility. The entire scene depicts a cross section through the cell, rendered in orthographic projection to allow comparison of sizes across the image, and employing sharp depth cueing to separate background from foreground.

Extended features such as the PopZ filament/IDR, chromosome, and peptidoglycan network are arranged entirely in the plane of the image, so that none are clipped. This choice is made to improve comprehension of the overall structure of these features, but may induce artifacts in the details of local arrangement and interactions. For example, the five PopZ filaments are arranged flat in the foreground layer, with the IDR all extending within this narrow slab of space. Three‐dimensional models of the condensate will be needed to explore more accurate arrangements of filaments within the condensate space.

Conformations of molecules are chosen to improve a high‐level understanding of the scene. PopZ IDRs are depicted as long, sinuous chains that interdigitate and fill space, whereas NMR structures of individual PopZ IDRs (PDB ID 6xry) show a more compact structure. Similarly, peptidoglycan and lipopolysaccharide chains are shown as mostly extended. In all cases, this gives an impression of the length of chains in relation to other molecules in the scene. These choices underscore the need for detailed mesoscale modeling to probe their 3D conformation within this crowded environment.

Colors and composition are chosen to highlight the central story told by the illustration: the structure and function of the condensate. The color palette highlights functional compartments of the cell, with microdomain in yellow and orange, cell wall in green, cytoplasm in blue, and nucleoid in magenta. Colors of individual molecules are consistent across the illustration, so, for example, soluble client proteins (orange) may be seen in different contexts in the microdomain and in the cytoplasm. Bright, warm colors are chosen for the microdomain and clients to focus attention on them. In previous bacterial illustrations, a visually stable composition was designed with the cell wall and flagellar motor at the top, enclosing the cytoplasm and nucleoid below. A reversed composition was employed in the *Caulobacter* illustration with the cell wall and flagellar motor at the bottom, microdomain centered, and nucleoid entering the frame from the top. This less‐stable composition was chosen intentionally to shift focus to the nucleoid and microdomain.

## 3 DISCUSSION

### 3.1 Science art collaboration

The current SciArt movement has goals as diverse as its community of scientists and artists, ranging from the free expression inherent in fine art (Gewin, 2021; Root‐Bernstein et al., 2011) to art that is conceived as a tool for scientific research and dissemination (Goodsell, 2021; Zhu & Goyal, 2019). The central goal in the work described here is to use traditional illustrative techniques to create an integrated conception of the state of the field—in this case, liquid–liquid phase separation in bacteria—for use in dissemination of results and outreach to other communities. However, the act of collaboration is arguably the most important aspect of this process. For example, this project provided the opportunity to formally gather and curate diverse information related to the molecular composition, abundance, and interactions of all components of condensate, often drawing on expert opinions from other researchers in the field. In essence, the illustration acts as a visual review of the field.

In these types of collaborations, it is important that there is a two‐way dialog throughout the process. Many challenges are addressed during this dialog. For example, early in planning, we chose to depict the flagellated pole in order to avoid the additional complexity of the stalk structure. Perhaps most importantly, this type of integrative illustration requires that decisions are made for aspects that are still under study, based on current hypotheses or intuition. As such, they represent a snapshot of the current state of the subject. Table 3 outlines several aspects of the microdomain structure that are still under study and the assumptions that we made when depicting these aspects in the illustration.

**TABLE 3:** Aspects of the illustration with limited available information.

Aspects with limited information	Assumptions in the illustration
Limited structural information for membrane‐bound clients	Combination of AlphaFold2 structures and UniProt annotations to depict domain structure
Site of PopZ interaction on client is not known for most clients (Table 2)	Arbitrary position chosen
Density of PopZ within condensate is not fully characterized	Condensate assigned typical concentration of prokaryotic cytoplasm
PopZ–PopZ interactions still under active study	Used trimer/hexamer/filament model from Lasker lab; additional filament/filament interactions, if they exist, not shown
Detailed confirmation of PopZ IDR within condensate not fully characterized	Assumed an extended conformation that improved clarity of the illustration
Interface of condensate with flagellar motor not characterized in detail	No specific interaction depicted
Complete composition of the PopZ microdomain is not known	Compiled a list of proteins residing within the microdomain based on current literature
Interface between microdomain and cytosol not fully characterized	Assumed PopZ IDRs are extended to bind soluble clients at the interface between microdomain and cytosol
Organization of membrane clients within the membrane is not fully characterized	Assumed uniform, random distribution of membrane‐bound clients

In addition, the choice of design and rendering often place restrictions on what can be shown. For example, the watercolor technique used here can show a scene several hundred nanometers wide while still making the individual macromolecules recognizable. The magnification, however, makes inclusion of small molecules, ions, and water unfeasible.

### 3.2 Implications to *Caulobacter* biology

This illustration provides an integrated view that for the first time integrates current knowledge into a single figure. Critical features of the domain are depicted, providing one conception of the interplay of PopZ, clients, flagellar motor, and the surrounding membrane and cytoplasm, all of which are consistent with currently available information. By doing so, the illustration provides a thinking tool for comprehension of the mesoscale properties and organization of the polar microdomain. For example, the choices made during design and rendering of the illustration pose several interesting mescoscale‐level questions. How are signals from the chemotaxis array transformed to the flagella motor through the PopZ microdomain? What is the arrangement of scaffolding proteins inside the condensate and how do they contribute to the arrangement of PopZ molecules? Is the ParB/*parS* interface with PopZ structurally and functionally different from the rest of microdomain? Similarly, is the interface between PopZ and the membrane different from the inner region? This illustration provides a visual conception for each of these questions, with the goal of helping researchers build a mental model of the system for hypothesis generation and further experimentation.

Research on cellular condensates is in a period of exponential growth, with many new examples being continually discovered. As is often true in new fields of inquiry, information is still fragmentary for many of these systems. The PopZ condensate is among the best studied of condensates, reaching a level where this type of integrative conception is possible without requiring an excessive level of artistic license. Looking forward, we expect that a similar approach will be applicable to many cellular systems. It is becoming apparent that they share many common features, being built around multivalent biomolecules like PopZ that include flexible intrinsically disordered segments in addition to folded regions to promote self‐association and interaction with client molecules.

### 3.3 Applications

The illustration is intended for general use in dissemination and outreach. For example, we have presented it as part of our professional presentations, such as the workshop “Re‐imagining a cellular space occupied by condensates” (October 2022, Salt Lake City, Utah). To promote widespread use, a full‐size tif file is freely available through a Creative Commons license at PDB‐101, the outreach portal for the RCSB Protein Data Bank (pdb101.rcsb.org) for use in presentations, lessons, and display in laboratories.

## 4 PROSPECTS

As mentioned in the introduction, we are increasingly using these types of illustrations at the beginning of large‐scale mesoscale modeling projects, as an approachable way to gather and curate information and identify key questions that could be addressed with full 3D modeling. The PopZ microdomain illustration underscores several aspects of the system as we move forward. First, it is one of the most well‐studied examples of a cellular condensate, so abundant data is available for many central aspects of the structure. Second, it is a relatively simple system with a manageable number of components, which should be amenable to detailed modeling. The illustration project also identifies several aspects where quantitative information is missing, which could be addressed through further experimental study and modeling. This includes the detailed nature of the PopZ filament, sites of PopZ interaction on client proteins and sites of interaction between neighboring PopZ filaments, the detailed structure of the membrane‐bound clients, and the in vivo density of PopZ in the condensate. Detailed 3D models will open the door to study of processes such as the diffusion and capture of clients within the PopZ matrix and the role of membrane‐bound clients in the localization of the condensate.

## 5 MATERIALS AND METHODS

### 5.1 Structural proteome and interactions

Previous studies have shown that PopZ includes Helix 1 near the N‐terminus, a central IDR rich in proline and negatively charged amino acids, and three closely spaced helices near the C‐terminus involved in oligomerization. These features are apparent in the AlphaFold2 model (AF‐Q9A8N4‐F1). AlphaFold predictions of the oligomerization domain yielded a parallel three‐stranded helical bundle, and docking of two trimers with Haddock (Honorato et al., 2021) yielded a hexamer model with parallel and slightly offset trimers. The width of the hexamer is consistent with the width of PopZ filaments observed in cryoelectron micrographs (Toro‐Nahuelpan et al., 2022). The filament is depicted as a speculative head‐to‐tail arrangement of hexamers, which results in a parallel double helix of trimers.

**FIGURE 4:**
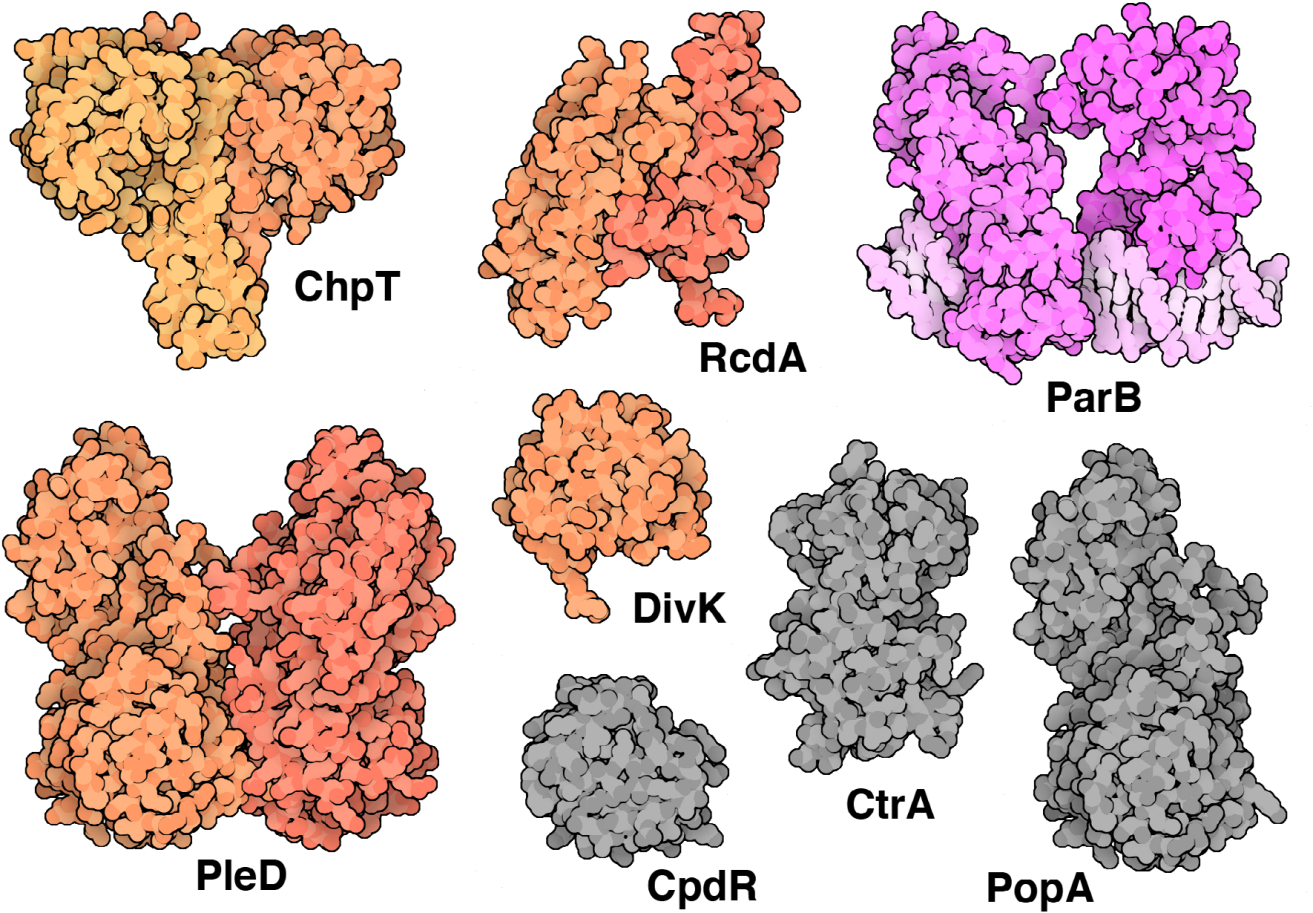
Experimental structures of soluble clients. Five structures are Caulobacter proteins (shown in color), and three are structures from related organisms or a homologous molecule in *Caulobacter* (shown in gray).

Soluble client proteins are based on experimental structures as shown in Figure 4. Structures of *Caulobacter* proteins were available for five soluble clients: PDB ID 4fpp (ChpT), 3ctw (RcdA), 6t1f (ParB and DNA), 1w25 (PleD), and 1mb3 (DivK). The ParB/DNA complex is missing the C‐terminal domain, which was depicted based on the AlphaFold2 structure (AF‐B8GW30‐F1). In addition, the structure Spo01 with DNA (PDB ID 4umk) was used to depict the open form of ParB. Homologous proteins were used for three soluble clients, all found using the SWISSMODEL link from the UniProt entry: 6tne (CpdR), 1ys6 (CtrA), and 6t1f (PopA).

**FIGURE 5:**
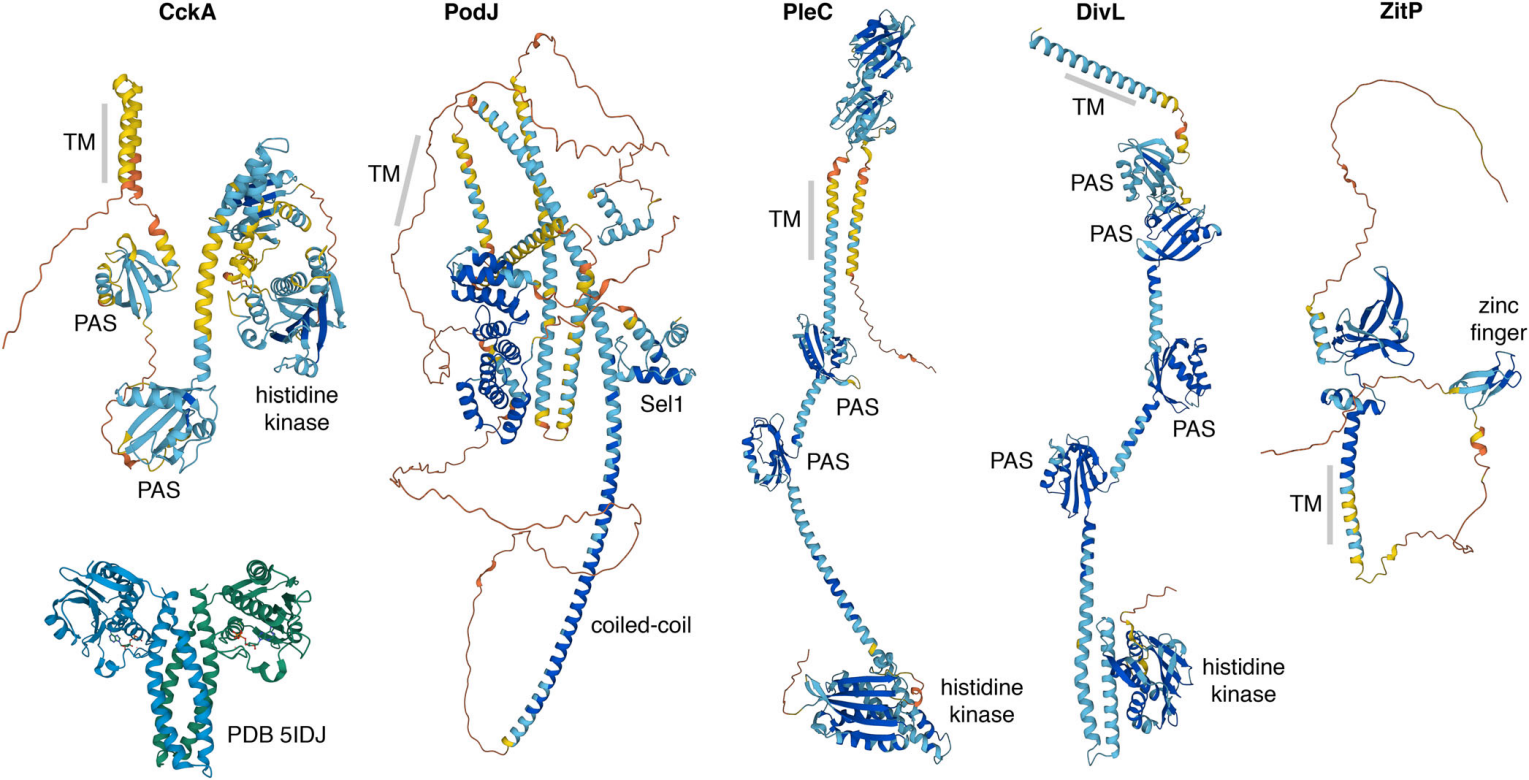
AlphaFold2 structures of membrane clients. Structures are colored with the standard scheme, with regions of high confidence in blue and regions of low confidence in yellow and red. The experimental structure of the histidine kinase domain of CckA is shown at lower left for comparison.

Membrane‐bound client proteins posed greater challenges. All are based on annotations in UniProt and AlphaFold2 predictions (Figure 5). CckA, PleC, and DivL all contain classic histidine kinase domains, which are dimeric in available crystal structures (5idj and 4q20), so the entire complex was treated as dimeric. CckA is additionally thought to assemble into tetramers (Mann & Shapiro, 2018). PodJ is annotated with a periplasmic Sel1 domain, which may mediate interaction with peptidoglycan, a transmembrane segment, a membrane‐proximal IDR, and three closely spaced coiled‐coil regions. It was treated as a dimer formed through a speculative long coiled coil. In addition, PodJ is known to be proteolyzed into a shorter form by removing the periplasmic domains (Lawler et al., 2006). Here, we treated all copies as the long form. ZitP was treated as a monomer based on the AlphaFold2 predicted structure.

Interactions with clients and PopZ (Table 2) were through the PopZ Helix 1. Interacting sites on clients are largely uncharacterized, apart from the zinc finger on ZitP (Bergé et al., 2016) and were depicted in speculative locations.

Structures for cytoplasmic and cell wall proteins were taken largely from a previous illustration of *Escherichia coli* (doi: 10.2210/rcsb_pdb/goodsell‐gallery‐028). Each protein was confirmed at UniProt to ensure presence in the *Caulobacter* genome. Differences with *E. coli* include absence of porins and lipoprotein LPP in the outer membrane and presence of many TonB‐linked transport complexes that span the cell wall. Specific proteins that are depicted were chosen by: (1) first choosing molecules from the 150 most abundant proteins in the ribosome profiling study (Schrader et al., 2016) and (2) filling remaining spaces with proteins from the *E. coli* illustration after verification for presence in the *Caulobacter* genome. The flagellar motor is based on structures from cryoelectron microscopy (Rossmann et al., 2020) and the S‐layer is taken from PDB entries 5n97 and 6t72.

### 5.2 Abundances

Relative copy numbers for microdomain proteins were obtained from ribosome profiling (Schrader et al., 2016), and the fraction of each protein that is found in the microdomain was estimated from fluorescence microscopy from multiple sources (see references in Table 1). To get absolute numbers for each molecule, we assumed that the microdomain was a sphere of radius 60 nm, and that the overall concentration is equivalent to the protein density in typical bacterial cytoplasm, 0.22 g/mL (bionumbers.hms.harvard.edu/bionumber.aspx?id=107695; Cayley et al., 1991). A total mass was calculated from the copy numbers, fractions, and molecular weights of microdomain proteins, revealing that these numbers needed to be divided roughly by a factor of 3 to yield an acceptable density. We then calculated the number of molecules depicted in the illustration by assuming that the foreground layer represented a central slice through this spherical condensate with a thickness of 10 nm.

### 5.3 Ultrastructure

The ultrastructure depicted in the painting is based on a cryoelectron micrograph (Dahlberg et al., 2020). Features taken from this micrograph include the spacing of membranes and S‐layer, location of chemosensory array relative to the microdomain, and distribution of ribosomes excluded by the microdomain. Size and spacing of PopZ filaments is based on micrographs (Toro‐Nahuelpan et al., 2022).

### 5.4 Illustration methods

The illustration was rendered as described previously (Goodsell, 2016). Briefly, style sheets for individual molecules were developed for all molecules and used to create a full sketch of foreground molecules, adding assemblies with long‐range coherence first (PopZ filament core, nucleoid, S‐layer, membranes, etc.), followed by large molecular assemblies (ribosomes, PopZ IDR), and finally filling remaining spaces with soluble components. The illustration is rendered in watercolor and ink using traditional color washes, starting with foreground and adding background molecules extemporaneously.

## AUTHOR CONTRIBUTIONS

**David S. Goodsell:** Conceptualization (equal); data curation (equal); funding acquisition (equal); investigation (equal); methodology (equal); software (equal); visualization (equal); writing – original draft (equal); writing – review and editing (equal). **Keren Lasker:** Conceptualization (equal); data curation (equal); funding acquisition (equal); investigation (equal); methodology (equal); software (equal); visualization (equal); writing – original draft (equal); writing – review and editing (equal).

## CONFLICT OF INTEREST STATEMENT

The authors declare no conflicts of interest.

## Data Availability

Included in the article: ”A full‐size digital file and key are available for unrestricted use at PDB‐101, the outreach portal of the RCSB Protein Data Bank at doi: 10.2210/rcsb_pdb/goodsell‐gallery‐046.”
